# Wound of the Heart

**Published:** 1841-08

**Authors:** 


*Wound of the Heart.*—Professor Malle relates in his *Clinique Chirurgicale de l' Hopital d' Instruction de Strasbourg*, the following remarkable case of wound of the heart.

"A soldier, ætat. 31, was amusing himself with a comrade in firing, when suddenly his friend's gun burst, and wounded him. He fell instantly in syncope, but soon regained his senses and complained of a severe pain behind the sternum. He was carried to the hospital, where he presented the following symptoms. About two inches on the inner side of the left nipple, between the sixth and seventh ribs, there was a small wound, which gave passage neither to blood nor to air. The chest was normally resonant beneath it; the patient had some bloody expectoration: the heart's motions were obscure and the pulse feeble; there was dyspnœa: the skin was cold, the face pale, and the patient felt as if he should faint. The surgeon in attendance regarding the greater part of these symptoms as the effect of the fright, ordered some simple means. Four hours after reaction took place, and the patient was bled to ten ounces, after which the expectoration of blood completely ceased. The pulsations of the heart, however, remained obscure, the pulse was small, and the sternal pain continued; but the face had regained its colour, the heat of the surface had returned, and the threatenings of fainting had passed away; he was bled again in the evening.

"Next day, the patient had passed a tranquil night; the pulse was fuller and regular, at 100; the face flushed but expressive of suffering; the pain behind the sternum continued; the ear distinguished at the part a kind of undulatory crepitation, something like that heard in a varicose aneurism; and there was a little crepitation in the left lung near the heart. Everywhere else the natural respiratory murmur was heard, though the dyspnœa continued; the horizontal position was irksome. The patient was again bled to ten ounces, and cupped over the heart. On each of the two following days the condition of the patient remained unaltered, and the same depletory means were repeated.

"On the fourth day after the accident he was better; he had slept four hours and had lost his faintness. He had less pain, there was no longer any râle, the pulse was less frequent and regular. The improvement continued during the succeeding days; the patient had some appetite and took some light food. The pain behind the sternum was almost gone; but there was still dulness in the precordial region; the pulse remained feeble, and he did not evidently gain strength. His apparent convalescence, though it advanced slowly, would probably have been more marked had it not been twice or three times interrupted by the patient's heat of temper and refusal of being restrained to perfect quietude. After each fit of anger, his cough became worse, the dulness in the precordial region more extensive, the pain at the sternum more acute, and the pulsations of the heart more obscure; and for every such aggravation of symptoms depletions were required and were generally efficient. He continued thus alternately improving and suffering from a return or aggravation of his first symptoms up to the forty-second day after the accident, when his condition was such as to permit a hope that his recovery would be permanent. At this time, however, he was seized, without any evident cause, with erysipelas of the left leg, with fever, &c.; his old symptoms returned with irrepressible violence, and he died on the 14th of May, having received the wound on the 28th of March.

"On examination the brain and the abdominal organs were found healthy. In the chest at the wounded part there was a cicatrix scarcely firm. The right lung presented on its anterior surface and near the heart, a small cicatrix, which was also discoverable under the corresponding portion posteriorly, proving that the lung had been perforated. It was also hepatized for about four inches, and united by some slight adhesions to the pleura. The pericardium was larger than usual, and at first appeared distended with liquid. It contained about five ounces of reddish sanies, and some fibrinous clots, two of which adhered to the heart. A foreign body was fixed in the left ventricle. On closer examination this was found to be a portion of the stock of the gun that had burst; it was situated at the front and about the middle third of the ventricle; its free extremity, which was about as large as a full-sized writing quill, projecting about ten lines; the cavity of the ventricle contained a very firm coagulum extending into the aorta. The piece of wood had traversed the left ventricle and the septum, and projected into the cavity of the right ventricle. Its direction was obliqely from without inwards and from below upwards; its form was somewhat triangular, tapering irregularly from the part that projected in front to that which traversed the septum. The internal surface of the heart was red, and in parts a little softened, especially near the apex; near the valves, on the contrary, the membrane appeared slightly hypertrophied."—*Brit. and For. Med. Rev.* July, 1840.

